# Analysis of ventilatory ratio as a novel method to monitor ventilatory adequacy at the bedside

**DOI:** 10.1186/cc12541

**Published:** 2013-02-27

**Authors:** Pratik Sinha, Nicholas J Fauvel, Pradeep Singh, Neil Soni

**Affiliations:** 1 Magill Department of Anaesthesia, Intensive Care Medicine and Pain Management, Chelsea and Westminster Hospital, 369 Fulham Road, London SW10 9NH, UK; 2 Department of Mathematics, Southeast Missouri State University, One University Plaza, Cape Girardeau, MO 63701, USA

## Abstract

**Introduction:**

Due to complexities in its measurement, adequacy of ventilation is seldom used to categorize disease severity and guide ventilatory strategies. Ventilatory ratio (VR) is a novel index to monitor ventilatory adequacy at the bedside. $\text{VR}=\left({\dot{V}}_{Emeasured}\times Pa_{CO_{2\;measured}}\right)/\left({\dot{V}}_{Epredicted}\times Pa_{CO_{2\;ideal}}\right)$. ${\dot{V}}_{Epredicted}$ is 100 mL.Kg^-1^.min^-1 ^and $Pa_{CO_{2\;ideal}}$ is 5 kPa. Physiological analysis shows that VR is influenced by dead space (V_D_/V_T_) and CO2 production $\left(\dot{V}CO_{2}\right)$. Two studies were conducted to explore the physiological properties of VR and assess its use in clinical practice.

**Methods:**

Both studies were conducted in adult mechanically ventilated ICU patients. In Study 1, volumetric capnography was used to estimate daily V_D_/V_T _and measure $\dot{V}CO_{2}$ in 48 patients. Simultaneously, ventilatory ratio was calculated using arterial blood gas measurements alongside respiratory and ventilatory variables. This data was used to explore the physiological properties of VR. In Study 2, 224 ventilated patients had daily VR and other respiratory variables, baseline characteristics, and outcome recorded. The database was used to examine the prognostic value of VR.

**Results:**

Study 1 showed that there was significant positive correlation between VR and VD/VT (modified r = 0.71) and $\dot{V}CO_{2}$ (r = 0.14). The correlation between VR and V_D_/V_T _was stronger in mandatory ventilation compared to spontaneous ventilation. Linear regression analysis showed that V_D_/V_T _had a greater influence on VR than $\dot{V}CO_{2}$ (standardized regression coefficient 1/1-V_D_/V_T_: 0.78, $\dot{V}CO_{2}$: 0.44). Study 2 showed that VR was significantly higher in non-survivors compared to survivors (1.55 vs. 1.32; *P *< 0.01). Univariate logistic regression showed that higher VR was associated with mortality (OR 2.3, *P *< 0.01), this remained the case after adjusting for confounding variables (OR 2.34, *P *= 0.04).

**Conclusions:**

VR is an easy to calculate bedside index of ventilatory adequacy and appears to yield clinically useful information.

## Introduction

Adequacy of ventilation is a crucial part of mechanical ventilation. Physiological dead space fraction (V_D_/V_T_) and CO_2 _production dictate the ventilatory demands of the body. In respiratory failure V_D_/V_T _is the most important factor that dictates ventilatory adequacy. Several studies have demonstrated the value of V_D_/V_T _in critically ill patients in both prognostication and disease progression in conditions such as acute respiratory distress syndrome (ARDS) [1-4]. Yet due to the complexity or equipment costs associated with its calculation, V_D_/V_T _is seldom measured in daily intensive care unit (ICU) practice [5,6]. The partial pressure of oxygen in arterial blood/fractional concentration of inspired oxygen (PaO_2_/FiO_2_) is a simple ratio that describes the adequacy of oxygenation and is widely used in daily practice despite being a poor predictor of outcome [7]. A similar quick reference tool does not exist for ventilation.

Recently, a simple bedside index of ventilatory adequacy has been described [8]. Ventilatory ratio (VR) is easy to calculate using minute ventilation and arterial partial pressure of carbon dioxide $\left(P_{CO_{2}}\right)$. VR is defined as:

(1) $$VR=\frac{{\dot{V}}_{E_{measured}}\times P_{a_{CO_{2\;measured}}}}{{\dot{V}}_{E_{predicted}}\times P_{a_{CO_{2\;predicted}}}}$$

where ${\dot{V}}_{Emeasured}$ is the measured minute ventilation (mL.min^-1^) and ${\dot{V}}_{Epredicted}$ is the predicted minute ventilation for the individual calculated as 100 mL.kg.min^-1^. $Pa_{CO_{2}measured}$ is the measured arterial $P_{CO_{2}}$; and $Pa_{CO_{2}predicted}$ is the ideal $Pa_{CO_{2}}$, defined as 5 kPa. VR is dimensionless and it is anticipated that a value approaching 1 would be 'normal'.

Analysis of VR shows it to be influenced by two variables that are seldom measured in critical care:

(2) $$VR=\frac{\dot{V}CO_{2_{actual}}}{E_{actual}}\times \frac{E_{predicted}}{\dot{V}CO_{2_{predicted}}}$$

where $\dot{V}CO_{2}$ is the volume of CO_2 _production per unit time and 'E', ventilatory 'efficiency', is described as the proportion of tidal volume that participates in gas exchange $\left(1-\frac{V_{D}}{V_{T}}\right)$. For a given individual, predicted values for 'E' and $\dot{V}CO_{2}$ are constant. Therefore equation 3 can be restated as:

(3) $$VR=\frac{\dot{V}CO_{2}}{1-V_{D}\;/)\;V_{T}}\times k$$

where *k *is specific to the individual. Inspection of equation 3 shows that VR would change as a result of a change in V_D_/V_T _or $\dot{V}CO_{2}$ or both.

Two studies were conducted with the following aims:

• Study 1: To evaluate the physiological properties of ventilatory ratio in a sample of mechanically ventilated critically unwell patients.

• Study 2: To evaluate ventilatory ratio in a general ICU population.

## Materials and methods

Both studies were conducted in patients aged > 18 years in a general medical and surgical ICU at Chelsea and Westminster Hospital. All patients were emergency admissions to the ICU.

### Study 1: Validation of physiological properties of ventilatory ratio

The study was approved by the local ethics committee (The National Hospital for Neurology and Neurosurgery and Institute of Neurology; REC Number: 10/H0716/16). Assent was obtained from family prior to recruitment to the study. Data were prospectively collected in patients with an admission PaO_2_/FiO_2 _ratio < 40 kPa. Initial measurements were made following stabilization of the patient and within the first 24 hours following admission to the ICU. Measurements were then made once daily until either six consecutive recordings or the patient had been weaned from the ventilator or had died, whichever was the shorter period. For each set of measurements a note was made of the mode of ventilation; mandatory (synchronized intermittent mandatory ventilation (SIMV) or bi-level positive pressure ventilation (BIPAP)) or spontaneous (assisted spontaneous breaths (ASB)). All modes of ventilation used non-bias flow triggering with the Dräger Evita XL ventilator (Dräger Medical, Lübeck, Germany).

As a form of internal validation, dead space fraction was estimated simultaneously using two different methods and measurement systems. The two methods: Douglas Bag and volumetric capnography showed good agreement and the findings of this comparative study is presented elsewhere [9]. For the study presented, V_D_/V_T _as calculated using volumetric capnography was used for analysis. V_D_/V_T _was calculated using the CO_2_SMO™ Plus capnograph (Novometrix Medical Systems, Wallingford, CT, USA). The CO_2_SMO™ Plus has a combined flow sensor and in-line capnograph that allows real-time simultaneous measurement of expiratory flow and expired CO_2 _concentration. The expired CO_2 _concentration is plotted against the expired volume to produce a simple single-breath test (SBTCO_2_) waveform. The software program Analysis Plus! for Windows (Novometrix Medical Systems, Wallingford, CT, USA) was used to estimate values for V_D_/V_T _using principles of areas under the SBTCO_2 _waveform [10].

$\dot{V}CO_{2}$ is the rate of CO_2 _elimination and, in a patient in steady state, this represents the rate of CO_2 _production. $\dot{V}CO_{2}$ was measured using CO_2_SMO™ Plus. Following the attachment of the capnograph, a period of 10 minutes was allowed without a change in ventilation or medical/nursing intervention for the patient to reach steady state. For patients without sedation, steady state was assessed visually. VR was calculated from measured $P_{CO_{2}}$ and ${\dot{V}}_{E}$ as recorded by the flow sensor of the ventilator. Predicted body weight was estimated using the formula set by the ARDS network group [11].

All readings were grouped together to examine the relationship between VR and $\dot{V}CO_{2}$ and V_D_/V_T_. It was anticipated that $\dot{V}CO_{2}$ would be greater in spontaneously ventilating patients [12]. Therefore for subgroup analysis, readings were categorized into groups depending on the mode of ventilation: mandatory or spontaneous.

### Study 2: Ventilatory ratio in an intensive care population

Permission was granted from the local ethics committee to anonymously analyze data from adult ICU charts between October 2008 and January 2011 and need for consent was waived. Complete data was available for 224 of the 312 patients. Missing patients were usually for those that were liberated from invasive ventilation within 24 hours.

Demographic data such as height, weight, gender, admission diagnosis, and past medical history were recorded. Mode of ventilation, arterial blood gas (ABG) results, and standard respiratory variables were recorded for all patients. These variables were used to calculate VR, PaO_2_/FiO_2 _ratio, and dynamic compliance. In addition APACHE II scores, mortality outcomes and ventilator days were also recorded. Data was recorded at admission and subsequently daily using the first arterial blood gas of the day until the patient died or was liberated from the ventilator.

For subgroup analysis, ICU patients were split into survivors and non-survivors. In addition the survivors were further subdivided into those with an admission VR of greater or equal to 1.4 and those with an admission VR less than 1.4. These subgroups was used assess the association of VR with prolonged mechanical ventilation.

To create a 'control group', respiratory data and ABG measurements were also collected on patients undergoing elective surgery. Data from patients requiring perioperative arterial cannulation were used for the study. All patients were undergoing abdominal surgery and were recruited as part of a larger study (REC Number: 10/H0720/24). Arterial blood gas was taken 20 minutes after induction of anesthesia and other respiratory variables were recorded simultaneously. All patients were extubated postoperatively and none required prolonged mechanical ventilation.

### Statistical analysis

Normally distributed data are expressed as mean (± standard deviation). Nonparametric data are expressed as median (interquartile range). Unpaired *t *test was used for intergroup comparison of means and Mann-Whitney test was used to compare medians for nonparametric data. Multiple comparisons of means across groups were made using one-way analysis of variance.

For Study 1, a modified Pearson's correlation coefficient as described by Stratton and colleagues [13] was obtained to study the association between VR and V_D_/V_T_. The method was used to correct bias in Pearson's correlation coefficient as a result of mathematical coupling due to $Pa_{CO_{2}}$ being used in the computation of both VR and V_D_/V_T_. Multiple regression analysis was performed to evaluate the effect of the variables 1/(1-V_D_/V_T_) and $\dot{V}CO_{2}$ on VR, and standardized coefficients were calculated to examine the influence of the two variables in this model. Logistic regression analysis was applied to examine predictive probabilities of VR for estimating ordinal groups of V_D_/V_T_.

For Study 2, univariate logistic regression analysis was used to calculate odds ratios for multiple respiratory variables to individually predict mortality. Multivariate logistic regression analysis was performed to examine the association of hospital mortality and VR and adjusted for confounding variables. Stepwise multiple logistic regression analysis was performed to determine the prognostic value of VR after adjustment for non-pulmonary outcome modifiers. Kaplan-Meier curves were constructed to examine the association of VR subgroups and ventilator days (admission to 30 days). Log-rank test was used to compare curves of the groups and hazard ratios were calculated.

Statistical software STATA/IC 11.1 (StataCorp, College Station, TX, USA) was used for data analysis. Graphs were constructed using Prism 5 for Mac OS X (Graphpad Software Inc., San Diego, CA, USA).

## Results

### Study 1: Validation of physiological properties of VR

Forty-eight patients (33 men and 15 women) were recruited to the study. In total 168 sets of daily readings were taken, of which 106 were from patients in a mandatory mode of ventilation and 62 from patients in spontaneous mode of ventilation. The median number of daily sets of readings per patient was 3 (range 2 to 6). The mean value of VR in all the readings was 1.97 (± 0.82). The baseline characteristics of this population are summarized in Table 1.

**Table 1:** Summary of baseline clinical characteristics of 48 patients from Study 1.

Variable	Data
Age (years)	61 ± 15
Weight (kg)	76 ± 15
Height (cm)	170 ± 9
Sex, Male/Female	33 (69)/15 (31)
*Admission diagnosis*	
LRTI/Exacerbation of COPD	15 (31)
Intra-abdominal pathology	14 (29)
Sepsis (non-respiratory)	9 (19)
Cardiac insufficiency	7 (15)
Miscellaneous	3 (6)
Underlying COPD/ILD	7 (16)
Developed ALI/ARDS	5 (10)
Outcome survivors/non-survivors	16 (33)/32 (67)

LRTI, lower respiratory tract infection; COPD, chronic obstructive pulmonary disease; ILD, interstitial lung disease; ALI, acute lung injury; ARDS, acute respiratory distress syndrome.

There was significant positive correlation between VR and V_D_/V_T _(modified r_p _= 0.71, *P *< 0.01) for the pooled data from all readings. In comparison to mandatory modes of ventilation, readings in spontaneous modes of ventilation showed weaker positive correlation between VR with V_D_/V_T _(mandatory modified r_p _= 0.76, *P *< 0.01; spontaneous modified r_p _= 0.67, *P *< 0.01). Table 2 summarizes the main differences in key respiratory variables between mandatory and spontaneous modes of ventilation. There was weak correlation between VR and $\dot{V}CO_{2}$ (r_p _= 0.14, *P *= 0.08). Linear regression analysis showed significant effect of V_D_/V_T _and $\dot{V}CO_{2}$ on VR $\left(\text{VR}=-0.05+1/\left(1-{\text{V}}_{\text{D}}/{\text{V}}_{\text{T}}\right)*0.29+\dot{V}CO_{2}*0.006;\;P<0.01\right)$. Standardized regression coefficients showed that in this model V_D_/V_T _exerted a greater influence on predicting $\text{VR}\left(1/\left(1-{\text{V}}_{\text{D}}/{\text{V}}_{\text{T}}\right)\beta =0.78,\dot{V}CO_{2}\;\beta =0.44\right)$.

**Table 2:** Mean values of measured and calculated variables in Study 1.

	Mandatory mode	Spontaneous mode	*P *value
VR	1.9 (0.8 - 5.4)	2.09 (0.9 - 4.2)	0.15
V_D_/V_T_	0.62 (± 0.13)	0.59 (± 0.12)	0.10
$\dot{V}CO_{2}$ (mL.min^-1^)	186 (± 57.3)	219 (± 68.2)	< 0.01
${\dot{V}}_{E}$ (mL.min^-1^)	10.3 (± 3.3)	13.1 (± 4.3)	< 0.01
$Pa_{CO_{2}}$ (kPa)	6.1 (± 1.34)	5.3 (± 1.51)	< 0.01

*P *values were derived using unpaired *t *test. The parentheses adjacent to VR represent range of values. All other parentheses are standard deviation. VR, ventilatory ratio; V_D_/V_T_, physiological dead space fraction; $\dot{V}CO_{2}$, CO_2 _production, ${\dot{V}}_{E}$, minute ventilation; PaCO_2_, partial pressure of carbon dioxide in arterial blood.

**Figure 1:**
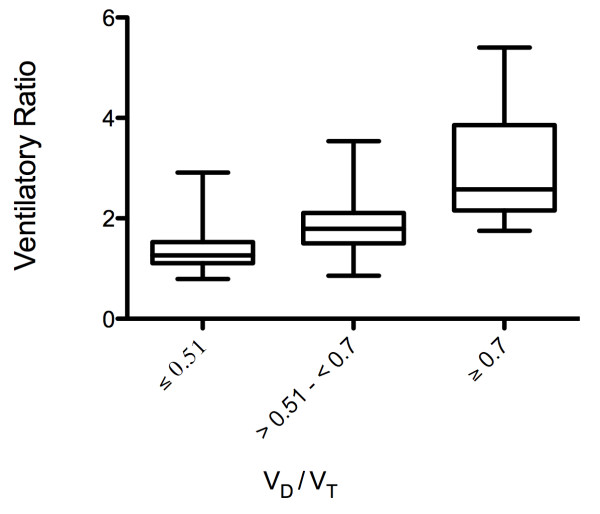
**Box-Whisker plot of VR with V_D_/V_T _categorized into subgroups according to severity of impairment**. (ANOVA *P *< 0.01). The results are from Study 1.

Figure 1 shows VR values in three different V_D_/V_T _ordinal subgroups. V_D_/V_T _was split into three categories according to clinical severity; normal: ≤ 0.51, moderate: > 0.51 to < 0.7, severe: ≥ 0.7 [2]. ANOVA showed that there were significant differences in the mean values of VR in the groups (*P *< 0.01). Concordance values obtained from logistic regression analysis were used to measure the association of predictive probabilities of VR and observed ordinal response of V_D_/V_T_. In mandatory modes of ventilation, the concordance between VR-predicted and observed ordinal groups of V_D_/V_T _was 84.7% and in spontaneous mode of ventilation the concordance was 80.9%.

### Study 2: Ventilatory ratio in an intensive care population

**Figure 2:**
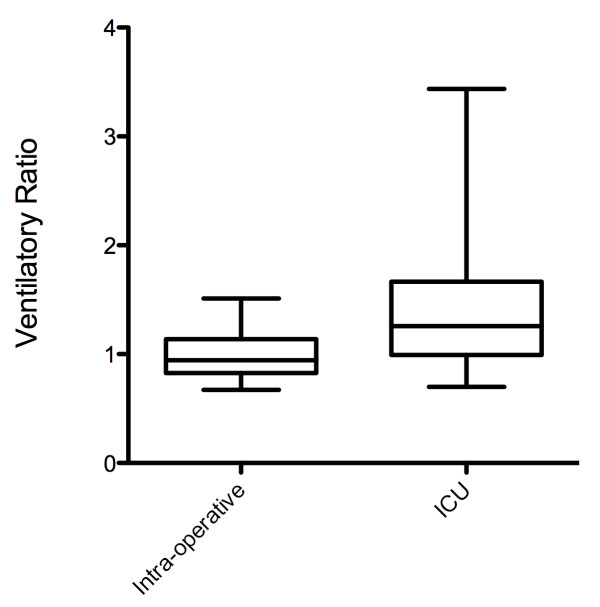
**Comparison of VR in intra-operative patients and ICU patients (Unpaired *t *test *P *< 0.01)**.

Data were captured on 224 ICU patients and 26 intraoperative patients. Mean values of VR were significantly higher in the ICU population compared to theater patients (ICU VR 1.4, CI 1.33 to 1.47; theater mean VR 0.99, CI 0.91 to 1.08; *P *< 0.01) (Figure 2). Subsequent results presented are of the ICU population only.

Survival outcomes were available for 214 patients. Ten patients were transferred to other ICU facilities and their outcome data was not available. Tables 3, 4 and 5 show comparative summaries of the baseline characteristics, and comparative demographic data and respiratory variables between survivors and non-survivors. A total of 154 patients survived to ICU discharge and 60 patients died. VR was higher on admission in non-survivors (survivors 1.32, CI 1.24 to 1.39; non-survivors 1.55, CI 1.39 to 1.70, *P *< 0.01). Univariate logistic analysis showed VR was an independent predictor of mortality on day 1 (OR 2.3, CI 1.3 to 4.1, *P *< 0.01), on day 2 (*n *= 194, OR 2.92, CI 1.41 to 6.01, *P *< 0.01), and day 3 (*n *= 152, OR 2.51, CI 1.30 to 4.9, *P *< 0.01). Table 6 summarizes the findings of univariate logistic analysis of respiratory variables on day 1. After adjusting for APACHE II score, peak inspiratory pressure, and positive end-expiratory pressure (PEEP), VR remained an independent predictor of mortality (OR 2.34, CI 1.03 to 4.08, *P *= 0.04).

**Table 3:** Admission diagnoses of ICU patients in Study 2.

Diagnosis	n (%)
**Gastrointestinal complications**	**60 (26.9)**
Intra-abdominal sepsis	20 (9.0)
Bowel perforation/Obstruction	21 (9.4)
Pancreatic/Hepatic failure	7 (3.1)
Postoperative complications	6 (2.7)
Gastrointestinal bleeding	6 (2.7)
**Respiratory insufficiency**	**47 (21.1)**
Lower respiratory tract infection	21 (9.4)
Exacerbation of COPD	10 (4.5)
ALI/ARDS	10 (4.5)
Acute pulmonary edema	4 (1.8)
Aspiration/Pneumonitis	3 (1.3)
**Cardiorespiratory arrest**	**37 (16.6)**
Cardiac arrest	35 (11.7)
Respiratory arrest	2 (0.8)
**Sepsis**	**16 (7.2)**
Urinary	5 (2.2)
Skin	5 (2.2)
Neutropenic	2 (0.8)
Unknown	4 (1.8)
**Miscellaneous**	**52 (23.3)**
Neurological complication	15 (6.7)
Poisoning/Overdose	13 (5.8)
Burns	12 (5.4)
Gynaecological problems	8 (3.6)
Postop cardiovascular monitoring	4 (1.8)
Multiorgan failure	4 (1.8)
Metabolic/Trauma	6 (2.7)

COPD, chronic obstructive pulmonary disease; ALI, acute lung injury; ARDS, acute respiratory distress syndrome.

**Table 4:** Comparison of demographic data between survivors and non-survivors.

	Survivors (*n *= 154)	Non-survivors (*n *= 60)	*P *values
Sex Male	82 (53.2%)	31 (51.7%)	0.84^§^
Age (years)	52.6 ± 19.3	62.2 ± 17.1	< 0.01
Weight (kg)	75.7 ± 18.5	72.1 ± 13.2	0.17
Height (cm)	170.8 ± 7.9	170.7 ± 8.1	0.88
APACHE II score	15.5 (11 - 19)	20 (19 - 23)	< 0.01*

*P *values are for unpaired *t *test for continuous variables; §chi-square test; *Mann-Whitney test.

**Table 5:** Comparison of respiratory variables at admission between survivors and non-survivors.

	Survivors (*n *= 154)	Non-survivors (*n *= 60)	*P *values
Minute ventilation (l.min^-1^)	7.7 ± 2.2	8.7 ± 3.3	< 0.01
PaCO_2 _(kPa)	5.5 ± 1.3	5.8 ± 1.6	0.22
Ventilatory ratio	1.32 ± 0.5	1.55 ± 0.6	< 0.01
PaO_2 _(kPa)	17.0 ± 6.4	18.5 ± 11.0	0.22
FiO_2_	0.54 ± 0.21	0.65 ± 0.23	< 0.01
PaO_2_/FiO_2 _ratio	36.2 ± 18	31.4 ± 17.6	0.08
PEEP (cmH_2_O)	5 (5 -10)	8 (5 - 10)	< 0.01*
Peak inspiratory pressure (cmH_2_O)	21 (16 - 26)	23 (18.25 - 30.75)	< 0.01*
Dynamic compliance	46.0 ± 29.9	38.6 ± 23.8	0.09

*P *values are for unpaired *t *test for continuous variables; *Mann-Whitney test. PaCO_2_, partial pressure of carbon dioxide in arterial blood; PaO_2_, partial pressure of oxygen in arterial blood; FiO_2_, fractional concentration of inspired oxygen; PEEP, positive end-expiratory pressure.

**Table 6:** Odds ratio derived from univariate analysis of individual respiratory variables on day 1 with mortality as the outcome (*n *= 223).

	Odds ratio	CI	*P *value
Ventilatory ratio	2.31	1.30 - 4.11	< 0.01
PaCO_2 _(kPa)	1.13	0.92 - 1.40	0.22
Minute ventilation (mL.min^-1^)	1.00	1.00 - 1.01	0.01
Peak inspiratory pressure (cmH_2_O)	1.07	1.02 - 1.11	< 0.01
PaO_2 _(kPa)	1.02	0.98 - 1.06	0.23
PaO_2_/FiO_2 _ratio	0.98	0.97 - 1.01	0.08
PEEP (cmH_2_O)	1.13	1.01 - 1.27	0.03
Dynamic compliance	0.99	0.97 - 1.00	0.1

PaCO_2_, partial pressure of carbon dioxide in arterial blood; PaO_2_, partial pressure of oxygen in arterial blood; FiO_2_, fractional concentration of inspired oxygen; PEEP, positive end-expiratory pressure.

**Figure 3:**
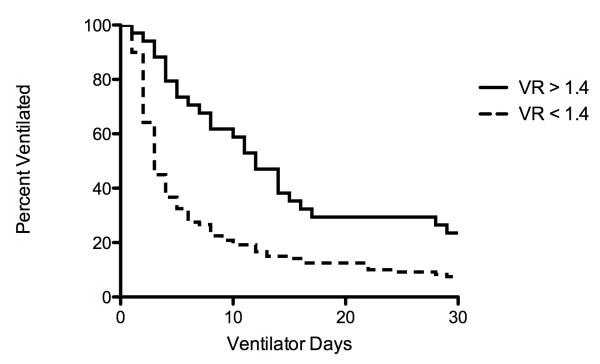
**Kaplan-Maier plot of ventilator days (28 days) in survivors**. The population of survivors was divided into those with VR ≥ 1.4 (*n *= 34) and those with VR < 1.4 (*n *= 120) at admission. Log-rank test showed the curves are significantly different (*P *< 0.01).

To further analyze the ability of VR at admission to predict disease severity, ventilator days were used as a surrogate. Among survivors, in 34 patients VR was greater or equal to 1.4 and in 120 patients VR was less than 1.4 at admission. Kaplan-Meier curves were constructed to examine ventilator days in the two groups with the end point being 28 days of mechanical ventilation (Figure 3). VR of greater that 1.4 at admission was more likely to result in prolonged ventilation (HR 2.2, CI 1.4 to 3.2, log-rank *P *< 0.01).

## Discussion

Observations in Study 1 showed that, as predicted by its physiological analysis, VR is influenced by both $\dot{V}CO_{2}$ and V_D_/V_T_. There was positive correlation between VR and dead space. In patients in mandatory modes of ventilation the correlation between VR and V_D_/V_T _was stronger. Linear regression analysis shows that in this population V_D_/V_T _was more influential than $\dot{V}CO_{2}$ in predicting VR. There was also good concordance between predicted ordinal groups of V_D_/V_T _using VR and the actual observed ordinal responses.

Study 2 showed that VR was significantly higher in ICU patients compared to elective intraoperative patients. VR was also significantly higher at admission in ICU non-survivors compared to survivors. Higher values of VR were associated with increased risk of mortality. This was the case after adjusting for confounding variables. In the group of patients that survived, a higher value of VR was associated with prolonged ventilation.

Ventilatory ratio is a marker of the lung's ability to cope with the ventilatory demands of the body. A rise in ventilatory demands would be as a result of either a rise in $\dot{V}CO_{2}$ or a rise in V_D_/V_T _or both. The findings in this study show that dead space changes resulted in a change in VR in 79% of the patients and in 65% of these cases this was exclusively due to changes in dead space. Ravenscraft and colleagues have similarly shown that in mechanically ventilated patients $\dot{V}CO_{2}$ is less of a contributor than V_D_/V_T _in rising ventilatory demands encountered during respiratory failure [14]. In sedated and ventilated patients it is anticipated that $\dot{V}CO_{2}$ will be relatively steady. In such patients the changes seen in VR in steady state are likely to be as a result of changes in V_D_/V_T_. The improved correlation of VR and in patients V_D_/V_T _in mandatory modes of ventilation substantiates this.

Although VR is able to predict ordinal groups of dead space, it is not an absolute measure of dead space. The premise of VR is that it should allow clinicians a relatively easy method of observing changes in ventilatory efficiency at the bedside. The use of predicted values for minute ventilation and $Pa_{CO_{2}}$ allows comparison within patients over time and across populations. The predicted values were set using old nomograms [15]. It likely that these predicted values overestimate the ventilatory needs of an individual. The values were pragmatically chosen at their current levels for ease of arithmetic calculations. Invariably, there will be some likely inaccuracies inherent to VR as a result of setting the predicted values to what they are. Nonetheless, a VR of 1 is likely to represent lungs and ventilatory settings that are able to clear CO_2 _efficiently. Conversely, a VR of 2 would be clear indication that the lungs in their current ventilatory setting are unable to cope with the ventilatory demands of the body. Given its physiological properties coupled with its strong association with outcome, VR is potentially a rapid, intuitive, and useful clinical tool to assess patients with respiratory failure.

There are several variables in the armamentarium of ICU physicians that are able to predict mortality. Features specific to VR make it a potentially attractive tool for physicians in addition to the other variables. To most physicians a composite function of two variables moving in opposite direction that tracks efficiency of CO_2 _clearance would be intuitively valuable and is currently not readily available. This is illustrated by the interaction of VR and the APACHE II score. As a physiological scoring system the APACHE II score is comprehensive and covers most aspects of impaired cardiovascular physiology [16]. Given VR remains an independent predictor of outcome after adjusting for APACHE II score suggests that it identifies an area of physiological dysfunction that is not engaged by the APACHE II score. In addition, current ICU practice relies on the PaO_2_/FiO_2 _ratio to categorize disease severity despite its inadequacies as a clinical tool being well documented. Not only is its value at admission as a predictor of outcome uncertain, there are also uncertainties surrounding its ability to categorize severity of disease, particularly in acute lung injury (ALI)/ARDS [17,18]. The results of this study show VR to be more closely associated with mortality than the PaO_2_/FiO_2 _ratio.

There are several limitations in this study. The data collected in Study 1 failed to capture data on patients that were spontaneously ventilating under heavy sedation. Most spontaneous ventilating readings were in patients that were being weaned from mechanical ventilation. The physiological behavior of VR in the former group of patients cannot be predicted from this study. In the clinical database, however, all patients were included and VR at admission was associated with outcome regardless of the mode of ventilation. Another shortcoming of the study is that the database is for all admissions to ICU and does not specifically examine patients with respiratory failure. It is in this group of patients that VR is anticipated to be most useful. Further studies are required to evaluate VR in respiratory failure patients.

## Conclusions

It stands to reason that patients with failure of oxygenation and ventilation (type II respiratory failure) would lead to a worse outcome than failure of oxygenation only (type I respiratory failure). In an era where alternative ventilatory strategies such as extracorporeal membrane oxygenation and extracorporeal CO_2 _removal exist, better and earlier recognition of impaired ventilation could lead to implementation of clinical practice that is less generic and more focused to underlying lung pathophysiology. VR is a quick bedside index that identifies such patients and the study shows that it is clinically useful in mechanically ventilated patients. VR is associated with outcome and provides clinicians with information that is currently not readily available.

## Key messages

• Ventilatory ratio is a simple bedside tool that allows quick assessment of ventilatory adequacy at the bedside.

• VR is influenced by dead space fraction and CO_2 _production. In the studied population, V_D_/V_T _was more closely associated with VR than CO_2 _production.

• VR shows good concordance with clinically relevant ordinal responses of V_D_/V_T_.

• VR was an independent predictor of mortality in a general ICU population. Higher VR was associated with worse outcome after adjusting for APACHE II score.

• VR has potential to identify patients with ventilatory failure and may be useful in identifying patients with worse outcome in ARDS.

## Abbreviations

ABG: arterial blood gas; ALI: acute lung injury; ARDS: acute respiratory distress syndrome; ASB: assisted spontaneous breaths; BIPAP: bi-level positive pressure ventilation; E: efficiency; ICU: intensive care unit; $Pa_{CO_{2}measured}$: measured arterial partial pressure of carbon dioxide; $Pa_{CO_{2}predicted}$: predicted arterial partial pressure of carbon dioxide; $P_{CO_{2}}$: partial pressure of carbon dioxide; PaO_2_/FiO_2_: partial pressure of oxygen in arterial blood/fractional concentration of inspired oxygen; PEEP: positive end-expiratory pressure; SIMV: synchronized intermittent mandatory ventilation; $\dot{V}CO_{2}$: carbon dioxide elimination/production; V_D_/V_T_: physiological dead space fraction; ${\dot{V}}_{E}$: expired minute ventilation; ${\dot{V}}_{Emeasured}$: expired measured minute ventilation; ${\dot{V}}_{Epredicted}$: expired predicted minute ventilation; VR: ventilatory ratio.

## Competing interests

The authors have no conflict of interests to declare.

## Authors' contributions

PS and NS were involved with study design, data collection, data analysis, and manuscript preparation. NJF was involved with design, analysis, and manuscript preparation. PS was involved with statistical design, analysis, and manuscript preparation. All authors have read and approved the final draft.
